# Utilizing the VirIdAl Pipeline to Search for Viruses in the Metagenomic Data of Bat Samples

**DOI:** 10.3390/v13102006

**Published:** 2021-10-06

**Authors:** Anna Y. Budkina, Elena V. Korneenko, Ivan A. Kotov, Daniil A. Kiselev, Ilya V. Artyushin, Anna S. Speranskaya, Kamil Khafizov, Vasily G. Akimkin

**Affiliations:** 1 FSBI Central Research Institute for Epidemiology of the Federal Service for Surveillance of Consumer Rights Protection and Human Wellbeing, 111123 Moscow, Russia; anna.y.budkina@gmail.com (A.Y.B.); lenakorneenko0@gmail.com (E.V.K.); ivan.kotov@phystech.edu (I.A.K.); hanna.s.939@gmail.com (A.S.S.); vgakimkin@yandex.ru (V.G.A.); 2 Moscow Institute of Physics and Technology, National Research University, 115184 Dolgoprudny, Russia; 3 I.M. Sechenov First Moscow State Medical University (Sechenov University), 119991 Moscow, Russia; neurolynx13@gmail.com; 4 Lomonosov Moscow State University, 119991 Moscow, Russia; sometyx@gmail.com

**Keywords:** viruses, NGS, bioinformatics, coronavirus, bats

## Abstract

According to various estimates, only a small percentage of existing viruses have been discovered, naturally much less being represented in the genomic databases. High-throughput sequencing technologies develop rapidly, empowering large-scale screening of various biological samples for the presence of pathogen-associated nucleotide sequences, but many organisms are yet to be attributed specific loci for identification. This problem particularly impedes viral screening, due to vast heterogeneity in viral genomes. In this paper, we present a new bioinformatic pipeline, VirIdAl, for detecting and identifying viral pathogens in sequencing data. We also demonstrate the utility of the new software by applying it to viral screening of the feces of bats collected in the Moscow region, which revealed a significant variety of viruses associated with bats, insects, plants, and protozoa. The presence of alpha and beta coronavirus reads, including the MERS-like bat virus, deserves a special mention, as it once again indicates that bats are indeed reservoirs for many viral pathogens. In addition, it was shown that alignment-based methods were unable to identify the taxon for a large proportion of reads, and we additionally applied other approaches, showing that they can further reveal the presence of viral agents in sequencing data. However, the incompleteness of viral databases remains a significant problem in the studies of viral diversity, and therefore necessitates the use of combined approaches, including those based on machine learning methods.

## 1. Introduction

Viruses can spread very swiftly, as humanity has seen time after time—and again in 2019, when SARS-CoV-2 struck and quickly disseminated internationally to cause the COVID-19 pandemic worldwide. Statistical simulations, albeit speculative, estimate that there may be over 320,000 different viruses [1], only about 200 of which have been reliably connected to human infections [2]. Although the number of known pathogens has steadily increased over the decades [3], emergence of novel viral infections has also been on the rise during recent years, for example, SARS [4,5], Ebola [6], MERS [7,8], Zika [9], and the recent COVID-19 outbreak [10]. Although it has not been reliably established [10,11], it is likely that the new coronavirus infection was transmitted to humans from bats, possibly through an intermediate host [12,13,14]. Thus, detailed studies of various types of biological samples from previously ignored locations and sources may contribute to early detection of new viral pathogens and better evaluation of the potential danger to human wellbeing.

High-throughput sequencing (NGS, next-generation sequencing) is a combination of technologies that allow for simultaneous reading of a large number of genomic fragments with high accuracy and reliability, to the point where their incorporation in routine medical practices is no longer seen as extravagant [15]. The idea of using NGS for the detection of viral pathogens was proposed about 10 years ago [15,16,17], soon after the appearance of sequencing techniques, and the concept has been gaining momentum rapidly as the technology advanced. The use of NGS to study viral diversity is at this time undoubtedly one of the most promising approaches, as suggested by its active application in such projects in recent years [18,19,20,21,22]. Researchers are primarily implementing metagenomic sequencing because it is a powerful tool for detecting a complete spectrum of viruses [23,24]. Due to its capabilities, the metagenomic approach has become more common in clinical and environmental studies [25,26].

However, despite the advantages mentioned above, metagenomic sequencing has apparent limitations inflicted by a plethora of factors: structures of pathogens, different efficiency of nucleic acid (NA) isolation from different viruses, GC content of their genomes, and others [27,28,29]. Separately, there is a question of determining the sensitivity of the method, as well as the problem of excess NA from host cells, bacteria, and fungi. However, the applicability of the approach has been repeatedly evaluated for the analysis of clinical samples, predicting valuable benefits for clinical trials [30,31,32].

Another issue at hand concerns data analysis. The pathogen content in a sample is not known in advance and can only be vaguely estimated by indirect evidence, for example, on the basis of the severity of the onset of the disease [33]. Therefore, evaluation of the minimum required number of sequencing reads per sample to ensure reliability of the readouts becomes a challenge and, in a way, a minefield. For instance, underestimation of this parameter results in a negligible and uninformative fraction of target sequences in the final fastq sequencing data file, falling far behind 1% [34,35]. Because the metagenomic approach clearly focuses on relative amounts of NA rather than on absolute values, setting the appropriate thresholds for quality estimation proves crucial for the reliability of the method.

One more problem in the search for viral pathogens is the bioinformatic analysis of NGS data, which is full of intricacies and tight spots. Pipelines for searching viral sequences in metagenomic data most often follow the following sequence: preprocessing, filtering, assembling, searching, and postprocessing [36]. In metagenomic sequencing, the overwhelming majority of reads refer to the genomes of the host and various incidental organisms. Therefore, taxon-based filtering saves processing time and computational resources by removing “unnecessary” sequences from the data before performing further steps. This approach has been ubiquitously introduced into processing pipelines, for example VirusSeeker [37], PAIPline [38], LAZYPIPE [39], and ViroMatch [40]. The search in the reference database can be conducted both for the raw reads and for the assembled contigs. Longer fragments can be assigned to the corresponding taxonomic group with greater accuracy. However, due to sequencing errors, chimeric sequences can sometimes be mistakenly assembled. Contig assembly is used in pipelines such as VIP [41] and virMine [42].

To determine the qualitative composition of metagenomic data, reads are classified on the basis of their attribution to a particular organism. The relative content of reads from a specific organism in the sample can also be assessed. The attribution of a read to a particular organism is often performed with similarity search, i.e., comparing acquired sequences against a reference database. BLAST is a popular tool for this task, but it is computationally wasteful to classify millions of reads in NGS data without prior filtering. To address this issue, more precise matches are searched. Examples of programs that exercise this approach include Kraken [43], Kraken2 [44], Centrifuge [45], Diamond [46], and megaBLAST [47]. These programs rapidly process input data; however, this is at the cost of sensitivity, as opposed to BLAST [48]. Additionally, the speed of the utilities decreases as sizes of reference databases expand—a problem which is yet to be solved. Another approach is the use of hidden Markov model profiles for searching in databases of protein domain profiles. This type of search allows for finding distant homologies and is resistant to sequence variability. An example of software that implements this approach is the HMMER tool [49], also as a part of VirSorter [50], virMine [42], and METAVIRAL SPADES [51].

The detection of viruses that significantly differ from known ones is a problem of scientific and clinical significance. One way of solving it concerns the use of machine learning. Models are trained on control sets of known viral sequences and afterwards applied to the unidentified sequences. The model predicts the likelihood that the reads belong to viral sequences. Random forest models were used in VirFinder [52], VirSorter2 [53], and MARVEL [53,54]. Deep learning models for virus identification were employed in Seeker [55], DeepVirFinder [56], ViraMiner [57], and DeePaC-vir [58].

However, many experimental laboratories that plan to use NGS in the analysis of clinical and environmental samples to search for fragments of viral genomes are bound to stumble upon a lack of qualified bioinformaticians or the necessity to develop software practically from scratch, which is unreasonable for episodic studies of individual samples. In this work, we attempted to create a convenient pipeline (VirIdAl) for analyzing NGS data, with the main task of identifying known viral pathogens. An important distinguishing feature of the algorithm is the ability to select those sequences that lack noticeable homology with reference sequences and require further investigation, for example, to verify their attribution to pathogenic microorganisms utilizing machine learning. The Materials and Methods section provides a detailed description of the pipeline operation and a brief manual. During its development, we set our main focus on the performance, since the alignment stage can take a very long time, becoming a critically limited resource during unforeseen infection outbreaks.

We also demonstrate the use of our new VirIdAl pipeline for analyzing NGS data obtained after processing biological material (feces) of bats collected in the Moscow region in 2015 and present our findings with regard to our original goal. All samples were found to contain genome fragments of the Coronaviridae and reads from other viral families. Importantly, several samples indicated the presence of MERS-like betacoronavirus, further indicating that bats are indeed a reservoir for many viral pathogens, some of which can be of particular interest for genomic epidemiology [59,60]. Thus, we believe that our new tool could be helpful for researchers using metagenomic NGS to study viral diversity, and especially for those who occasionally use sequencing to detect viral agents, including potentially new ones.

## 2. Materials and Methods

### 2.1. Pipeline Description

At the first stage of the pipeline, the input fastq files validity is verified using the fastQValidator tool (https://github.com/statgen/fastQValidator, accessed on 23 April 2021). If the input files contain paired-end reads, the fastp tool [61] is used to merge the overlapping reads, minimizing the amount of processed data and increasing the sequence length for a more accurate and specific search. Fastp is used then for quality filtering to trim adapters, discard reads with an average “Phred quality score” below a given value, remove low-quality nucleotides from the 5’- and 3’-ends of the sequence applying a sliding window, and discard sequences shorter than the specified length. The file obtained after filtering “fastq” is sequentially aligned to a given list of genomes, which allows for quick filtering out of host sequences and prokaryotic sequences that are uninformative for further targeted search. Alignment is performed using the Bowtie 2 utility [62] in default mode. Reads that are precisely aligned with the given genomes are attributed to the host and, therefore, discarded.

The resulting fastq file is then converted to the fasta format, and the sequences in the new file are clustered using the vsearch utility [63] with the cluster-fast option and with the given identity. The resulting “centroid” sequences, i.e., representative sequences in each cluster, are passed downstream of the pipeline.

The “search phase”, which consumes most of the processing time, accurately identifies known (or similar to known) viral sequences from the sequencing data. The search for known viral sequences consists of two sub-steps: (1) alignment of sequences from the input files to the nucleotide and amino acid sequences of viruses, and (2) alignment of the potential viral sequences identified at the first stage to the full NCBI nt and nr databases. At the first step, the high sensitivity of the search allows for the selection of the sequences that most likely belong to viruses. This stage reduces the number of sequences that will be aligned to the full non-redundant NCBI nt and nr databases, thus significantly reducing the duration of the next step. A search in exclusively virus-related databases proves much faster than scanning against the whole nt/nr. At the first step, a search is carried out using the megablast utility [47] and the Diamond utility [46] in the “sensitive” mode. A specific E-value threshold can be set for the search. The higher the threshold value, the higher the sensitivity of the assay, which allows for the detection of distant homology between sequences, although at the cost of reduced specificity.

**Figure 1:**
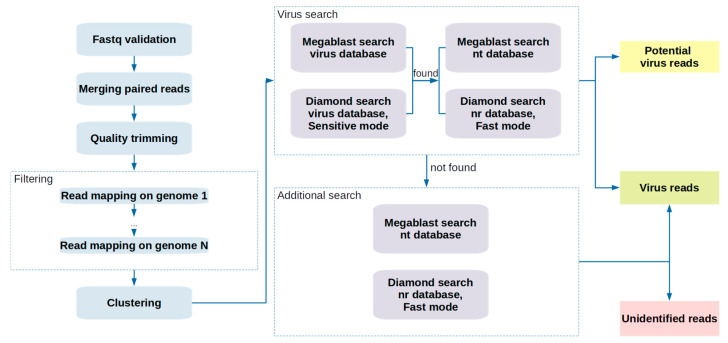
Visual representation of the VirIdAl bioinformatics pipeline for analyzing high-throughput sequencing data for searching DNA fragments of viral pathogens (virus reads), as well as detection of sequences that cannot be identified only on the basis of homology with known genomes (unidentified reads).

At the second step, the alignment is performed using the megablast tool for nucleotide (nt) sequences and Diamond in “fast” mode for amino acid (nr) sequences. The NCBI nt and nr databases contain a high number of nucleotide and amino acid sequences from a wide variety of organisms, and this number is constantly growing. In this step, the E-value threshold is adjustable: low threshold provides results with fewer false positives, and the search identifies sequences with close homology. For each sequence, the most probable search result is then selected (i.e., with the lowest E-value). On the basis of these results, it is possible to select those sequences that have been identified as viral. The flow chart in Figure 1 depicts the steps of the pipeline.

### 2.2. Computation Details

In order to demonstrate the usage of the pipeline, we processed 23 datasets obtained from 13 bat samples (see experimental details below). The preprocessing of the datasets included quality control, merging, filtering, and clustering stages. The quality control stage consists of removing adapters, low complexity sequences (complexity below 30%), and sequences with an average PHRED quality score of less than 20 and with a length shorter than 36. The sequences were trimmed at the start and the end with a sliding window size of 9 bp and an average quality score of 20.

The filtering stage included mapping the sequences to a set of bacterial and archaeal genomes and the *Pipistrellus pipistrellus* reference genome (https://www.ncbi.nlm.nih.gov/genome/?term=txid59474, accessed on 25 March 2021). The genomes of bacteria and archaea were downloaded from the RefSeq database on 19 March 2021 and 23 March 2021, respectively, using genome_updater (https://github.com/pirovc/genome_updater, accessed on 19 March 2021). Complete genome, chromosome, scaffold, and contig assembly level sequences were included in the filtering dataset. Clustering was performed with the default vsearch options and a “0.9” identity threshold.

The virus identification procedure included the virus search stage and the validation of the results at the additional search stage. Virus hits from both analyses were added to the results. For the first step of the virus search stage, the E-value threshold 1 × 10^−3^ was used both for megablast and Diamond search in the virus nucleotide databases (RefSeq and GenBank) downloaded from https://www.ncbi.nlm.nih.gov/labs/virus/vssi/#/ on 25 March 2021. Virus protein database featured RefSeq sequences and was downloaded from https://ftp.ncbi.nlm.nih.gov/refseq/release/viral/ on 25 March 2021. During the second step of the virus search stage, the sequences were aligned to the NCBI nt and nr databases. E-value threshold 1 × 10^−10^ was used both for nucleotide and protein search. The additional search was performed using the 1 × 10^−10^ E-value threshold for megablast and Diamond search in the nt and nr databases.

### 2.3. Sample Collection, Storage, and Library Preparation

In 2015 fecal samples were collected from 13 bats of the following species: *Myotis dasycneme* (*n* = 2), *Myotis daubentonii* (*n* = 2), *Myotis brandtii* (*n* = 1), *Nyctalus noctula* (*n* = 2), and *Pipistrellus nathusii* (*n* = 5), inhabiting on the territory of the Zvenigorodsky district of the Moscow region (Sharapovskoe forestry). For one sample, species of the source bat could not be established. The fecal pellets content was mainly of Arthropoda chitin remnants and pellets matched in size, form, and consistence to pellets of small Vespertilionid family bat. Fecal samples were placed in a transport solution with a mucolytic agent (TSM; Amplisens, Russia) and were stored at −70 °C until the experiments began.

RNA extraction was performed using the QIAamp Viral RNA Mini Kit (Qiagen, Hilden, Germany) and QIAcube automatic station (Qiagen, Hilden, Germany) according to the manufacturer’s protocol with elution in 50 uL of AVE buffer (elution buffer). At the same time, viral RNA extraction kits are also known to be applicable for metagenomic analysis of the DNA viruses [64]. Reverse transcription was performed using Reverta-L (Amplisens, Moscow, Russia) to obtain the first strand. The following library preparation steps were performed using the NEBNext Ultra II Directional RNA Library Prep Kit for Illumina (NEB, Ipswich, MA, USA) according to the manufacturer’s protocol, excluding the first strand cDNA synthesis. We replaced the NEB First Strand cDNA Synthesis step with Reverta-L (Amplisens, Moscow, Russia) since the RNA was expected to be degraded during transportation. Adaptor Ligation and PCR Enrichment of Adaptor Ligated DNA were performed using NEBNext Multiplex Oligos for Illumina (NEB, Ipswich, MA, USA). Paired-end sequencing was performed on the Illumina HiSeq 1500 platform using HiSeq Rapid SBS Kit v2 (500 cycles) and HiSeq PE Rapid Cluster Kit v2. The raw fastq files were uploaded to NCBI sequence read archive and are available under the following IDs: SRR15508011 (Bat sample number-2), SRR15508267 (22), SRR15508152 (6), SRR15524530 (20), SRR15524163 (16), SRR15524477 (19), SRR15525307 (21), SRR15526222 (27), SRR15533060 (30), SRR15533116 (31), SRR15534018 (36), SRR15540904 (33), SRR15540905 (23).

## 3. Results

**Figure 2:**
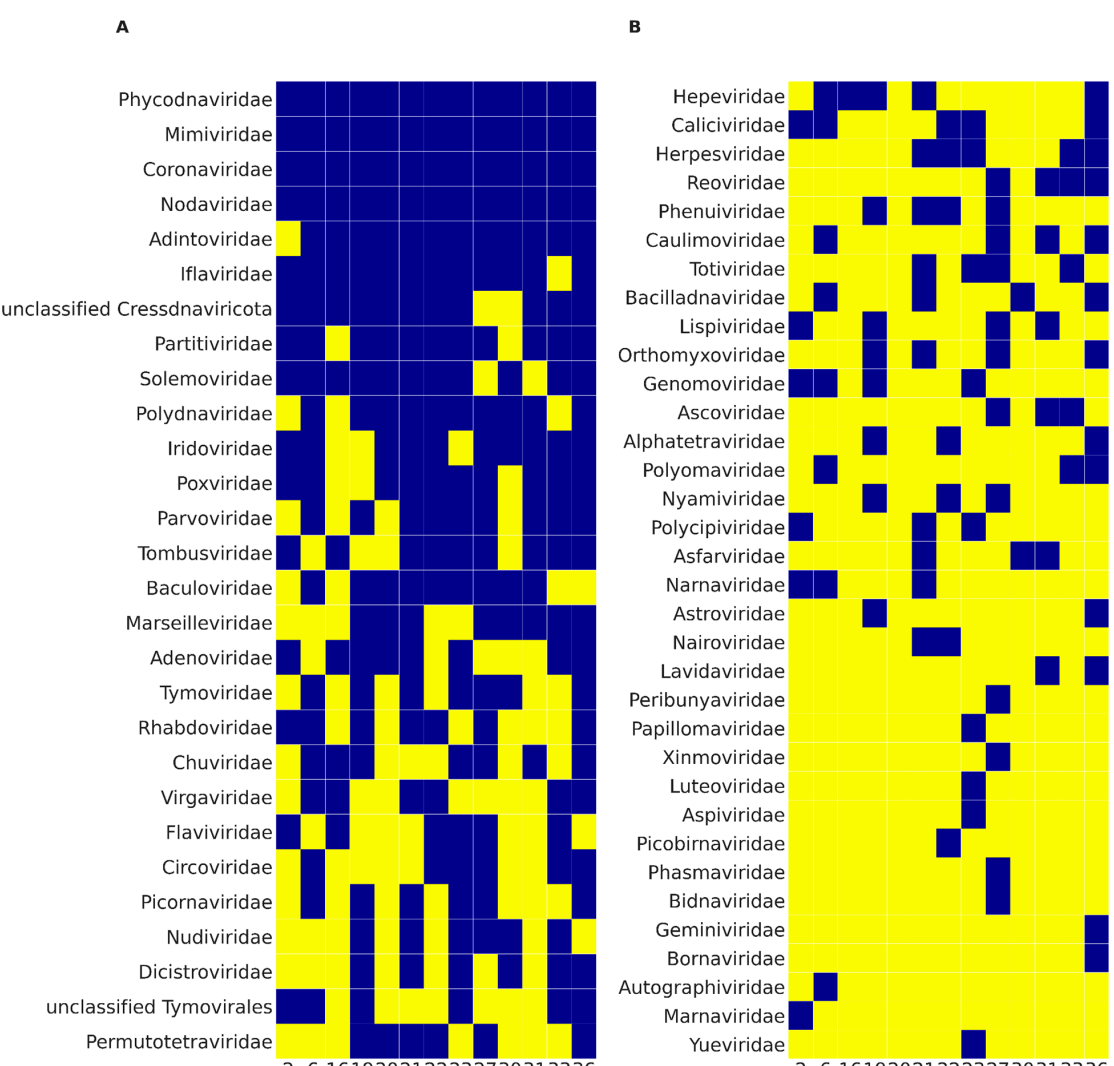
Presence of reads of viral genomes in bat samples in terms of the results of total sequencing. (**A**) Viral families in which reads were detected in at least six independent samples. (**B**) Viral families in which reads were detected in five or fewer samples. Blue rectangles correspond to the presence of the related viruses in the sample, yellow rectangles correspond to the absence. Numbers at the bottom represent bat samples.

We performed high-throughput sequencing of 13 samples of bat feces collected in 2015 in the Moscow region and applied our newly developed analysis pipeline VirIdAl to detect and identify viral DNA sequences. Taxonomic classification was assigned to each sequence identified by the pipeline as viral. Retroviridae, Metaviridae, and phage-related sequences were discarded from further analysis. Figure 2 illustrates the presence of viruses belonging to specific families found in the samples. The majority of the identified viral sequences can be assigned to viruses hosted by bats, insects, protozoa, and plants, with bat and insect viruses being the most prevalent in the readouts. Viral reads that belong to the Iflaviridae family, hosted by insects, were found in almost all samples analyzed in this study. The sequences of this family have been identified as *Iflaviridae sp*. and as various types of Iflaviruses, such as *Infectious flacherie virus*, *Sacbrood virus*, *Soybean thrips iflavirus*, and *Culex Iflavi-like virus*. Adintoviridae family viruses were found in almost all samples, and the most common viruses were *Spodoptera moth adintovirus*, *Megastigmus wasp adintovirus*, *Bos-associated insect adintovirus*, *Drosophila-associated adintovirus*, and *Ladona dragonfly adintovirus*. Bracoviruses from the Polydnaviridae family were present in the vast majority of samples; *Cotesia sesamiae Mombasa bracovirus* and *Cotesia vestalis bracovirus* were the most common across all samples. Among the Poxviridae family viruses discovered, viruses hosted and transmitted by insects predominate, such as *Betaentomopoxvirus* and *Leporipoxvirus*. Among the viruses of the Iridoviridae family in the samples, the most common sequences are detected as *Armadillidium vulgare iridescent virus* and *Invertebrate iridescent virus*. Another family of insect viruses, Baculoviridae, was found in more than half of the samples, and the most common viruses were *Lambdina fiscellaria nucleopolyhedrovirus* and *Mamestra configurata nucleopolyhedrovirus A*. Nodaviridae family viruses were associated with bats (*Bat nodavirus*) or insects (*Flock House virus*, *Newington virus*, *Black beetle virus*).

All samples were also shown to contain Phycodnaviridae viruses hosted by algae. The most common alignments for Phycodnaviridae were *Phaeocystis globosa virus*, *Yellowstone Lake phycodnavirus*, *Organic Lake phycodnavirus*, and *Paramecium bursaria Chlorella virus*. Protozoa viruses of the Mimiviridae family were found in all samples. *Klosneuvirus KNV1*, *Bodo saltans virus*, *Cafeteria roenbergensis virus BV-PW1*, and *Hyperionvirus* sp. were the most often detected members of this family across all samples. More than half of the samples contained Marseilleviridae viral sequences. CRESS-DNA viruses from Cressdnaviricota phylum were present in almost all samples.

**Figure 3:**
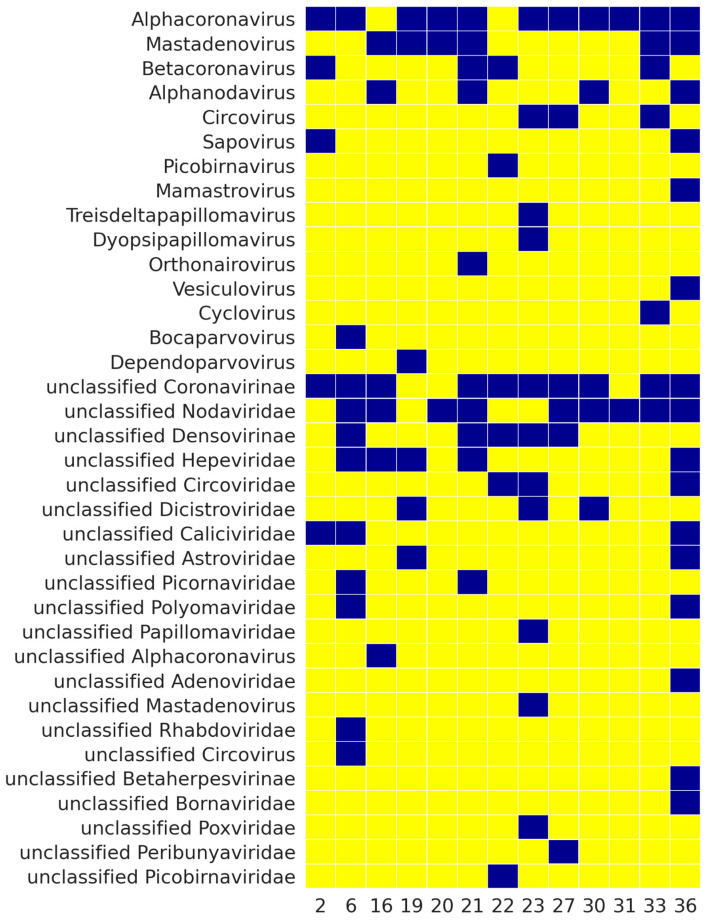
Presence of reads of viral fragments in bat samples based on sequencing results. Only those viral genera which bats can host are presented. Blue rectangles correspond to the presence of the related viruses in the sample, yellow rectangles correspond to the absence. Numbers at the bottom represent bat samples.

As follows from Figure 2, all the files contain reads from the Coronaviridae family, which is not surprising as bats are well-known reservoirs of coronaviruses [65,66]. Figure 3 shows the viral genera found in the analyzed samples that bats can host. Coronavirus sequences were discovered in all the analyzed samples. Alphacoronavirus reads were detected in all the samples, except for 16 and 22, but sample 16 contained unclassified Coronavirinae sequences.

The contigs in all samples were assembled with megahit [67]. The contig sequences were searched in the nt database using megablast and in the nr database using Diamond. The contigs aligned to Alphacoronavirus and Betacoronavirus sequences were selected for further analysis. Alphacoronavirus contigs were assembled in samples 2, 21, 23, 31, and 33. The longest contig of 28,448 bp was obtained from sample 21 and matched best MN065811.1 *Bat alphacoronavirus strain BtCoV/008_16/M.bra/FIN/2016*, a viral genome deposited in 2016 by a research group from Finland [59], with 82.7% identity. Most of the contigs from samples 23 and 33 had the top hit at MZ218060.1 *Bat coronavirus isolate BtCoV/7542-55/P.pyg/DK/2014* with percent identity higher than 80%, including the longest contig of 4276 bp long from sample 23. The contigs from sample 31 had the highest score alignments with *Alphacoronavirus Bat-CoV/P.kuhlii/Italy/206679-3/2010* (MH938450.1), *Bat coronavirus isolate BtCoV/B40-5/P.pyg/DK/2013* (MN482242.1), *Alphacoronavirus Bat-CoV/P.kuhlii/Italy/3398-19/2015* (NC_046964.1), and *BtNv-AlphaCoV/SC2013* (KJ473809.1). The contigs of sample 2 matched *Bat coronavirus isolate BtCoV/7542-55/P.pyg/DK/2014* (MZ218060.1) with percent identities of 93.3% and 91.2%, *Bat coronavirus isolate Anlong-57* (KY770851.1) with percent identities of 83.1% and 80.0%, and with *Bat coronavirus isolate BtCoV/21164-6-alt/M.dau/DK/2015* (MZ218052.1) with percent identity of 86.6%.

Betacoronavirus genera sequences were found in samples 2, 21, 22, and 33. Sample 2 contained a single nucleotide sequence classified as Betacoronavirus and was therefore excluded from further analysis. Interestingly, the vast majority of Betacoronavirus sequences were identified as the Middle East respiratory syndrome-related coronavirus. Contigs identified as Middle East respiratory syndrome-related coronavirus by searching the nt database using megablast or nr database by Diamond were selected. The length of the largest MERS-related viral contig was 2762 bp (sample 22).

In each sample, the largest number of contigs had the best match with sequences MG596803.1 and MG596802.1 related to the *Middle East respiratory syndrome-related coronavirus isolate Bat-CoV/H.savii/Italy/206645-40/2011* and *Middle East respiratory syndrome-related coronavirus isolate Bat-CoV/P.khulii/Italy/206645-63/2011*, respectively. The percent identity of these alignments varied from 74.5% to 87.8%. In sample 21, MG596803.1 had the highest alignment score for 3 of the 16 contigs. Other alignments included *Middle East respiratory syndrome-related coronavirus isolate NL140455*, complete genome (MG987421.1) with 88.9% identity; *Middle East respiratory syndrome-related coronavirus isolate Hu/Riyadh-KSA-13984/2016*, complete genome (MG011342.2) with 88.6% identity; and *Bat coronavirus Vs-CoV-1* genomic RNA, nearly complete genome (LC469308.1) with 83.4% identity. Besides MG596803.1 and MG596802.1, the contigs from sample 22 had the highest score alignments to *Middle East respiratory syndrome-related coronavirus isolate NL140455*, complete genome (MG987421.1) with 80.7% and 87.9% identities; *Hypsugo bat coronavirus HKU25 isolate YD131305*, complete genome (KX442564.1) with 78.1% identity; and *Bat coronavirus Vs-CoV-1* genomic RNA, nearly complete genome (LC469308.1) with 87.3% and 86.9% identities. Two out of six obtained contigs in sample 33 had the highest score alignment with MG596802.1. Other highest score alignments included *Middle East respiratory syndrome-related coronavirus strain Hu/Riyadh-KSA-18012493/2018*, complete genome (MK462249.1) with 88.7% identity, and *Middle East respiratory syndrome-related coronavirus strain Camel/Oman_1_2015*, complete genome (KY673149.1) with 76.4% identity.

Two reads from sample 6 were identified as the L-protein (large structural protein, a part of the RNA polymerase) sequence of *Eptesicus fuscus rhabdovirus* (QPO14166.1) using diamond tool with percent identities of 72.7 and 68.0, respectively. Megablast was then used to re-scan these reads without applying any E-value threshold. The identified sequences were separately aligned to the nt database and ViPR Rhabdoviridae nucleotide database using megablast. ViPR Rhabdoviridae database was downloaded from https://www.viprbrc.org on 29 September 2021 and included all the available sequences from the Rhabdoviridae family. The nt database search revealed that the first sequence shared 87.8% identity with the *Rabies lyssavirus* genomes MK598338.1, MK598339.1, MK598340.1, and MK598341.1. The best matches in the ViPR database search were *Rabies virus* (KU055561) and *Rabies lyssavirus* (KX148160, MK598338-MK598342) with 88.0% identity. At the same time, megablast did not return any results for the second sequence. The two reads were then translated into the amino acid sequences, and the corresponding proteins were scanned using hmmscan and Pfam profile-HMM database. Hmmscan identifies both sequences as Mononegavirales RNA dependent RNA polymerase with E-values 2.9 × 10^−13^ and 1 × 10^−9^, which is consistent with the megablast and diamond search results.

We also noticed that a significant part of the reads was not assigned to any taxon by the pipeline from the analysis of the results. Table 1 shows the number and percentage of sequences that have not been identified as belonging to known cellular organisms or viruses. While fine-tuning the pipeline parameters would possibly increase the number of reads attributed to known organisms, an inexorable rise in false positives would likely follow. This result indicates rather clearly that, firstly, the used sequence databases are far from complete, and secondly, other methods of analysis are definitely required to detect very distant homologies. In addition, if the research aims to study the diversity of viral agents, methods based on machine learning, an actively developing approach [55,56,57,68] can also be useful. As a preliminary attempt, we used the LSTM model of DeePaC-vir tool [58] to check if at least some of the remaining reads could belong to virus families. The default DeePaC-vir model was trained to detect human-infecting virus sequences; nonhuman virus sequences were used as a negative dataset. However, it also separates human viruses from the host sequences and other organisms. Furthermore, some viruses can be hosted by both humans and bats, including Betacoronaviruses. The analysis was carried out on samples 21, 22, and 33, since MERS-related virus sequences were identified in these samples. First, DeePaC-vir was applied to the two groups of sequences: the reads that were not assigned to any organism by the pipeline and the reads that were identified by the pipeline as Betacoronavirus sequences. The distributions of the proportion of sequences depending on the likelihood that they are viral for these two groups are shown in Figures S1 and S2. As can be seen from the shape of the distribution, most sequences from the first group have a low probability of being classified as viral. At the same time, a relatively small proportion of reads were still designated as likely belonging to viruses. The DeePaC-vir score distribution for Betacoronavirus reads is very different: a much larger portion of sequences receive a high likelihood score than the undefined sequences set.

Unidentified sequences with scores > 0.5 in the LSTM model, i.e., that are likely to be viral, were selected and re-analyzed using hmmscan search from HMMER 3.3.2 (http://hmmer.org/, accessed on 10 June 2021). The nucleotide sequences were translated into the amino acid sequences by the transeq program [69]. Six sequences were obtained using all the reading frames, and the longest protein sequence was selected. The resulting protein sequences were compared against the Pfam profile-HMM database [70]. Virus protein sequences were selected among the obtained results, excluding phage sequences. These sequences were re-scanned using megablast and diamond with a default E-value threshold; the identified sequences were discarded from the results. Table 2 shows the number of sequences obtained at each stage of this procedure.

Virus sequences that had been identified by hmmscan with the E-value below 0.01 were selected. Coronavirus protein sequences, such as Coronavirus nucleocapsid, Coronavirus M matrix/glycoprotein, Coronavirus non-structural protein NS4, and Coronavirus nonstructural protein NS1, were found in samples 21 and 22. Among the found sequences, proteins of viruses that can be hosted by bats were identified, such as Mamastrovirus p20 protein (PF12285.10), Adenovirus E3A (PF05248.14), Picornavirus 2B protein (PF01552.19), Parvovirus non-structural protein NS1 (PF01057.19), and Amdovirus non-structural protein (PF12475.10). Further, protein sequences of viruses that can be hosted by plants were detected, such as Geminivirus putative movement protein (PF01708.18), Geminivirus AC4/5 conserved region (PF08464.12), Potato leaf roll virus readthrough protein (PF01690.19), Luteovirus coat protein (PF00894.20), Carlavirus coat (PF08358.12), and Benyvirus P25/P26 protein (PF05744.13). Insect virus protein sequences were also detected: Baculoviridae P74 N-terminal (PF08404.12), Baculovirus polyhedron envelope protein, PEP, C terminus (PF04513.14), and Baculovirus 11 kDa family (PF06143.13).

Therefore, among the reads not determined by standard alignment methods, the sequences of viral proteins were found. These results demonstrate that “traditional” alignment-based methods do not identify viral sequences with 100% sensitivity. Other approaches for detecting remote homology can be used to discover sequences that are highly distinct from the reference sequences in the reference database. This is especially important for viruses due to their high mutation rate. One example of such a method is HMMER, which searches sequences against hidden Markov models profiles database [71]. Secondly, machine learning techniques can be employed to identify viral sequences, including deep learning models. The usage of various approaches can increase the efficiency of the search.

## 4. Discussion

The development of highly sensitive computational pipelines for detection of poorly described or entirely unknown viral sequences in biological samples represents an important milestone in epidemiological research. Frequently, upon the rise of an epidemic, the investigation into a pathogen’s genetic and molecular features lags substantially behind the rates of its dissemination. The loss of precious time results in an untimely and only a moderately effective response. Likewise, conventional screening methods quickly become outdated because a mere broad pinpointing of a pathogen’s taxonomy fails to supply crucial data, such as markers of drug resistance, and other intricate details. Importantly, precise mapping of a pathogen’s migration routes can only be done with high-resolution tools, capable of detecting minute changes in its genome for tracking its circulation. To date, only a single family of approaches grants all the important insights with high reproducibility and reliability, namely, high-throughput sequencing, enhanced with highly versatile and sensitive computational software.

In this article, we presented our new computational pipeline for detection and identification of viral reads in NGS data. As indicated in the introduction, the lack of skilled bioinformaticians and the need for software development often restrict the widespread use of NGS for detecting viruses and studying viral diversity. The new pipeline implements standard alignment tools to search for viral sequences. The number of false positives is reduced by running a two-stage search—first in the databases of virus sequences, then in the full nt and nr databases. Our software also finds sequences that were not recognized by the alignment tools to pass them to further stages of analysis. Additionally, the pipeline considers the genomes of well-described organisms for preliminary data filtering, which can contribute to a significant reduction in the amount of data for downstream processing. Multiple analysis parameters can be tailored to increase the accuracy of viral NA detection. VirIdAl is available at https://github.com/budkina/VirIdAl (accessed on 1 October 2021) and can be built as a Docker image. Additionally, the repository contains instructions for loading and formatting databases for searching.

Having provided a detailed description of the pipeline, we also demonstrated its application using the data obtained by sequencing bat feces collected in the Moscow region in 2015. A number of different viruses have been detected, including the alpha and beta coronaviruses of bats. Among them, a MERS-like coronavirus was identified, which shares 74.5–87.8% identity with the known *Middle East respiratory syndrome-related coronavirus isolate Bat-CoV/H.savii/Italy/206645-40/2011* (MG596803.1) genome. These results again corroborate that bats indeed harbor a plethora of viral pathogens, including coronaviruses. In addition to identifying known viruses and viruses genetically resembling them, separate files can be created as the pipeline’s output that would contain sequences that lack significant homology with any of the sequences stored in the databases. For these reads, we used LSTM-based methods for sequence prioritization in an attempt to identify more distant homologies by applying hidden Markov models for those identified as likely viral by specialized software. As a result, homology was found with several viral proteins, including those that are coronavirus-related. We hope that our pipeline will be helpful to biologists who use NGS to identify known and emerging viral pathogens. We believe that new methods and approaches, in addition to alignment-based, could prove beneficial for identifying new viruses that exhibit high sequence variability.

## Figures and Tables

**Table 1:** The numbers of sequences in clustered fasta files and the numbers of sequences that were not assigned to any taxon.

Sample ID	Number of Sequences in Clustered Fasta Files	Number of Unidentified Sequences	Percentage of Unidentified Sequences (%)
2	1,496,002	892,558	59.7
6	738,554	363,592	49.2
16	653,809	119,176	18.2
19	906,791	404,517	44.6
20	538,581	145,623	27.0
21	764,442	428,544	56.1
22	777,210	272,237	35.0
23	1,466,914	711,736	48.5
27	2,245,717	1,498,197	66.7
30	651,436	197,040	30.3
31	830,335	313,898	37.8

**Table 2:** The numbers of sequences in clustered fasta files and the numbers of sequences that were not assigned to any taxon.

Sample ID	Unidentified Sequences	Sequences with DeePaC-Vir Score > 0.5	Virus Sequences Identified by HMMER Hmmscan Only, E-Value < 10
21	428,544	63,969	177
22	272,237	38,413	82
33	482,033	35,361	69

## Data Availability

VirIdAl is available at https://github.com/budkina/VirIdAl accessed on 1 October 2021 and can be built as a Docker image. Additionally, the repository contains instructions for loading and formatting databases for searching.

## Supplementary Materials

The following are available online at https://www.mdpi.com/article/10.3390/v13102006/s1, Figure S1: Distribution of the DeePaC-vir LSTM model scores assigned to unclassified sequences in samples 21 (A), 22 (B), and 33 (C). Figure S2: Distribution of the DeePaC-vir LSTM model scores assigned to Betacoronavirus sequences from samples 21 (A), 22 (B), and 33 (C).

## References

[1] Anthony S.J., Epstein J.H., Murray K.A., Navarrete-Macias I., Zambrana-Torrelio C.M., Solovyov A., Ojeda-Flores R., Arrigo N.C., Islam A., Ali Khan S. (2013). A Strategy to Estimate Unknown Viral Diversity in Mammals. MBio.

[2] Woolhouse M., Scott F., Hudson Z., Howey R., Chase-Topping M. (2012). Human Viruses: Discovery and Emergence. Philos. Trans. R. Soc. Lond. B Biol. Sci..

[3] Jones K.E., Patel N.G., Levy M.A., Storeygard A., Balk D., Gittleman J.L., Daszak P. (2008). Global Trends in Emerging Infectious Diseases. Nature.

[4] Sørensen M.D., Sørensen B., Gonzalez-Dosal R., Melchjorsen C.J., Weibel J., Wang J., Jun C.W., Huanming Y., Kristensen P. (2006). Severe Acute Respiratory Syndrome (SARS): Development of Diagnostics and Antivirals. Ann. N. Y. Acad. Sci..

[5] Stadler K., Masignani V., Eickmann M., Becker S., Abrignani S., Klenk H.-D., Rappuoli R. (2003). SARS—Beginning to Understand a New Virus. Nat. Rev. Microbiol..

[6] Park D.J., Dudas G., Wohl S., Goba A., Whitmer S.L.M., Andersen K.G., Sealfon R.S., Ladner J.T., Kugelman J.R., Matranga C.B. (2015). Ebola Virus Epidemiology, Transmission, and Evolution during Seven Months in Sierra Leone. Cell.

[7] Danielsson N., Catchpole M., ECDC Internal Response Team (2012). Novel Coronavirus Associated with Severe Respiratory Disease: Case Definition and Public Health Measures. Euro Surveill..

[8] Corman V.M., Eckerle I., Bleicker T., Zaki A., Landt O., Eschbach-Bludau M., van Boheemen S., Gopal R., Ballhause M., Bestebroer T.M. (2012). Detection of a Novel Human Coronavirus by Real-Time Reverse-Transcription Polymerase Chain Reaction. Euro Surveill..

[9] Metsky H.C., Matranga C.B., Wohl S., Schaffner S.F., Freije C.A., Winnicki S.M., West K., Qu J., Baniecki M.L., Gladden-Young A. (2017). Zika Virus Evolution and Spread in the Americas. Nature.

[10] Huang C., Wang Y., Li X., Ren L., Zhao J., Hu Y., Zhang L., Fan G., Xu J., Gu X. (2020). Clinical Features of Patients Infected with 2019 Novel Coronavirus in Wuhan, China. Lancet.

[11] Segreto R., Deigin Y. (2021). The Genetic Structure of SARS-CoV-2 Does Not Rule out a Laboratory Origin: SARS-CoV-2 Chimeric Structure and Furin Cleavage Site Might Be the Result of Genetic Manipulation. Bioessays.

[12] Burki T. (2020). The Origin of SARS-CoV-2. Lancet Infect. Dis..

[13] Andersen K.G., Rambaut A., Lipkin W.I., Holmes E.C., Garry R.F. (2020). The Proximal Origin of SARS-CoV-2. Nat. Med..

[14] Piplani S., Singh P.K., Winkler D.A., Petrovsky N. (2021). In Silico Comparison of SARS-CoV-2 Spike Protein-ACE2 Binding Affinities across Species and Implications for Virus Origin. Sci. Rep..

[15] Metzker M.L. (2009). Sequencing Technologies—The next Generation. Nat. Rev. Genet..

[16] Adams I.P., Glover R.H., Monger W.A., Mumford R., Jackeviciene E., Navalinskiene M., Samuitiene M., Boonham N. (2009). Next-Generation Sequencing and Metagenomic Analysis: A Universal Diagnostic Tool in Plant Virology. Mol. Plant Pathol..

[17] Radford A.D., Chapman D., Dixon L., Chantrey J., Darby A.C., Hall N. (2012). Application of next-Generation Sequencing Technologies in Virology. J. Gen. Virol..

[18] Jansen S.A., Nijhuis W., Leavis H.L., Riezebos-Brilman A., Lindemans C.A., Schuurman R. (2020). Broad Virus Detection and Variant Discovery in Fecal Samples of Hematopoietic Transplant Recipients Using Targeted Sequence Capture Metagenomics. Front. Microbiol..

[19] Chiu C.Y. (2013). Viral Pathogen Discovery. Curr. Opin. Microbiol..

[20] Giallonardo F.D., Töpfer A., Rey M., Prabhakaran S., Duport Y., Leemann C., Schmutz S., Campbell N.K., Joos B., Lecca M.R. (2014). Full-Length Haplotype Reconstruction to Infer the Structure of Heterogeneous Virus Populations. Nucleic Acids Res..

[21] De Vries J.J.C., Brown J.R., Couto N., Beer M., Le Mercier P., Sidorov I., Papa A., Fischer N., Oude Munnink B.B., Rodriquez C. (2021). Recommendations for the Introduction of Metagenomic next-Generation Sequencing in Clinical Virology, Part II: Bioinformatic Analysis and Reporting. J. Clin. Virol..

[22] Kiselev D., Matsvay A., Abramov I., Dedkov V., Shipulin G., Khafizov K. (2020). Current Trends in Diagnostics of Viral Infections of Unknown Etiology. Viruses.

[23] Gu W., Miller S., Chiu C.Y. (2019). Clinical Metagenomic Next-Generation Sequencing for Pathogen Detection. Annu. Rev. Pathol..

[24] Alquezar-Planas D.E., Mourier T., Bruhn C.A.W., Hansen A.J., Vitcetz S.N., Mørk S., Gorodkin J., Nielsen H.A., Guo Y., Sethuraman A. (2013). Discovery of a Divergent HPIV4 from Respiratory Secretions Using Second and Third Generation Metagenomic Sequencing. Sci. Rep..

[25] Venter J.C., Remington K., Heidelberg J.F., Halpern A.L., Rusch D., Eisen J.A., Wu D., Paulsen I., Nelson K.E., Nelson W. (2004). Environmental Genome Shotgun Sequencing of the Sargasso Sea. Science.

[26] Mulcahy-O’Grady H., Workentine M.L. (2016). The Challenge and Potential of Metagenomics in the Clinic. Front. Immunol..

[27] McLaren M.R., Willis A.D., Callahan B.J. (2019). Consistent and Correctable Bias in Metagenomic Sequencing Experiments. Elife.

[28] Boers S.A., Jansen R., Hays J.P. (2019). Understanding and Overcoming the Pitfalls and Biases of next-Generation Sequencing (NGS) Methods for Use in the Routine Clinical Microbiological Diagnostic Laboratory. Eur. J. Clin. Microbiol. Infect. Dis..

[29] Chen Y.-C., Liu T., Yu C.-H., Chiang T.-Y., Hwang C.-C. (2013). Effects of GC Bias in next-Generation-Sequencing Data on de Novo Genome Assembly. PLoS ONE.

[30] Kustin T., Ling G., Sharabi S., Ram D., Friedman N., Zuckerman N., Bucris E.D., Glatman-Freedman A., Stern A., Mandelboim M. (2019). A Method to Identify Respiratory Virus Infections in Clinical Samples Using next-Generation Sequencing. Sci. Rep..

[31] Choi S.-H., Hong S.-B., Ko G.-B., Lee Y., Park H.J., Park S.-Y., Moon S.M., Cho O.-H., Park K.-H., Chong Y.P. (2012). Viral Infection in Patients with Severe Pneumonia Requiring Intensive Care Unit Admission. Am. J. Respir. Crit. Care Med..

[32] Datta S., Budhauliya R., Das B., Chatterjee S., Vanlalhmuaka, Veer V. (2015). Next-Generation Sequencing in Clinical Virology: Discovery of New Viruses. World J. Virol..

[33] Hijano D.R., Brazelton de Cardenas J., Maron G., Garner C.D., Ferrolino J.A., Dallas R.H., Gu Z., Hayden R.T. (2019). Clinical Correlation of Influenza and Respiratory Syncytial Virus Load Measured by Digital PCR. PLoS ONE.

[34] Allen U.D., Hu P., Pereira S.L., Robinson J.L., Paton T.A., Beyene J., Khodai-Booran N., Dipchand A., Hébert D., Ng V. (2016). The Genetic Diversity of Epstein-Barr Virus in the Setting of Transplantation Relative to Non-Transplant Settings: A Feasibility Study. Pediatr. Transplant..

[35] Matranga C.B., Andersen K.G., Winnicki S., Busby M., Gladden A.D., Tewhey R., Stremlau M., Berlin A., Gire S.K., England E. (2014). Enhanced Methods for Unbiased Deep Sequencing of Lassa and Ebola RNA Viruses from Clinical and Biological Samples. Genome Biol..

[36] Nooij S., Schmitz D., Vennema H., Kroneman A., Koopmans M.P.G. (2018). Overview of Virus Metagenomic Classification Methods and Their Biological Applications. Front. Microbiol..

[37] Zhao G., Wu G., Lim E.S., Droit L., Krishnamurthy S., Barouch D.H., Virgin H.W., Wang D. (2017). VirusSeeker, a Computational Pipeline for Virus Discovery and Virome Composition Analysis. Virology.

[38] Andrusch A., Dabrowski P.W., Klenner J., Tausch S.H., Kohl C., Osman A.A., Renard B.Y., Nitsche A. (2018). PAIPline: Pathogen Identification in Metagenomic and Clinical next Generation Sequencing Samples. Bioinformatics.

[39] Plyusnin I., Kant R., Jääskeläinen A.J., Sironen T., Holm L., Vapalahti O., Smura T. (2020). Novel NGS Pipeline for Virus Discovery from a Wide Spectrum of Hosts and Sample Types. Virus Evol..

[40] Wylie T.N., Wylie K.M. (2021). ViroMatch: A Computational Pipeline for the Detection of Viral Sequences from Complex Metagenomic Data. Microbiol. Resour. Announc..

[41] Li Y., Wang H., Nie K., Zhang C., Zhang Y., Wang J., Niu P., Ma X. (2016). VIP: An Integrated Pipeline for Metagenomics of Virus Identification and Discovery. Sci. Rep..

[42] Garretto A., Hatzopoulos T., Putonti C. (2019). virMine: Automated Detection of Viral Sequences from Complex Metagenomic Samples. PeerJ.

[43] Wood D.E., Salzberg S.L. (2014). Kraken: Ultrafast Metagenomic Sequence Classification Using Exact Alignments. Genome Biol..

[44] Wood D.E., Lu J., Langmead B. (2019). Improved Metagenomic Analysis with Kraken 2. Genome Biol..

[45] Kim D., Song L., Breitwieser F.P., Salzberg S.L. (2016). Centrifuge: Rapid and Sensitive Classification of Metagenomic Sequences. Genome Res..

[46] Buchfink B., Xie C., Huson D.H. (2015). Fast and Sensitive Protein Alignment Using DIAMOND. Nat. Methods.

[47] Zhang Z., Schwartz S., Wagner L., Miller W. (2000). A Greedy Algorithm for Aligning DNA Sequences. J. Comput. Biol..

[48] Ye S.H., Siddle K.J., Park D.J., Sabeti P.C. (2019). Benchmarking Metagenomics Tools for Taxonomic Classification. Cell.

[49] Mistry J., Finn R.D., Eddy S.R., Bateman A., Punta M. (2013). Challenges in Homology Search: HMMER3 and Convergent Evolution of Coiled-Coil Regions. Nucleic Acids Res..

[50] Roux S., Enault F., Hurwitz B.L., Sullivan M.B. (2015). VirSorter: Mining Viral Signal from Microbial Genomic Data. PeerJ.

[51] Antipov D., Raiko M., Lapidus A., Pevzner P.A. (2020). Metaviral SPAdes: Assembly of Viruses from Metagenomic Data. Bioinformatics.

[52] Ren J., Ahlgren N.A., Lu Y.Y., Fuhrman J.A., Sun F. (2017). VirFinder: A Novel K-Mer Based Tool for Identifying Viral Sequences from Assembled Metagenomic Data. Microbiome.

[53] Guo J., Bolduc B., Zayed A.A., Varsani A., Dominguez-Huerta G., Delmont T.O., Pratama A.A., Gazitúa M.C., Vik D., Sullivan M.B. (2021). VirSorter2: A Multi-Classifier, Expert-Guided Approach to Detect Diverse DNA and RNA Viruses. Microbiome.

[54] Amgarten D., Braga L.P.P., da Silva A.M., Setubal J.C. (2018). MARVEL, a Tool for Prediction of Bacteriophage Sequences in Metagenomic Bins. Front. Genet..

[55] Auslander N., Gussow A.B., Benler S., Wolf Y.I., Koonin E.V. (2020). Seeker: Alignment-Free Identification of Bacteriophage Genomes by Deep Learning. Nucleic Acids Res..

[56] Ren J., Song K., Deng C., Ahlgren N.A., Fuhrman J.A., Li Y., Xie X., Poplin R., Sun F. (2020). Identifying Viruses from Metagenomic Data Using Deep Learning. Quant. Biol..

[57] Tampuu A., Bzhalava Z., Dillner J., Vicente R. (2019). ViraMiner: Deep Learning on Raw DNA Sequences for Identifying Viral Genomes in Human Samples. PLoS ONE.

[58] Bartoszewicz J.M., Seidel A., Renard B.Y. (2021). Interpretable Detection of Novel Human Viruses from Genome Sequencing Data. NAR Genom. Bioinform..

[59] Kivistö I., Tidenberg E.-M., Lilley T., Suominen K., Forbes K.M., Vapalahti O., Huovilainen A., Sironen T. (2020). First Report of Coronaviruses in Northern European Bats. Vector Borne Zoonotic Dis..

[60] Li B., Si H.-R., Zhu Y., Yang X.-L., Anderson D.E., Shi Z.-L., Wang L.-F., Zhou P. (2020). Discovery of Bat Coronaviruses through Surveillance and Probe Capture-Based next-Generation Sequencing. mSphere.

[61] Chen S., Zhou Y., Chen Y., Gu J. (2018). Fastp: An Ultra-Fast All-in-One FASTQ Preprocessor. Bioinformatics.

[62] Langmead B., Salzberg S.L. (2012). Fast Gapped-Read Alignment with Bowtie 2. Nat. Methods.

[63] Rognes T., Flouri T., Nichols B., Quince C., Mahé F. (2016). VSEARCH: A Versatile Open Source Tool for Metagenomics. PeerJ.

[64] Zhang D., Lou X., Yan H., Pan J., Mao H., Tang H., Shu Y., Zhao Y., Liu L., Li J. (2018). Metagenomic Analysis of Viral Nucleic Acid Extraction Methods in Respiratory Clinical Samples. BMC Genom..

[65] Calisher C.H., Childs J.E., Field H.E., Holmes K.V., Schountz T. (2006). Bats: Important Reservoir Hosts of Emerging Viruses. Clin. Microbiol. Rev..

[66] Banerjee A., Kulcsar K., Misra V., Frieman M., Mossman K. (2019). Bats and Coronaviruses. Viruses.

[67] Li D., Liu C.-M., Luo R., Sadakane K., Lam T.-W. (2015). MEGAHIT: An Ultra-Fast Single-Node Solution for Large and Complex Metagenomics Assembly via Succinct de Bruijn Graph. Bioinformatics.

[68] Ma H., Tan T.W., Ban K.H.K. (2021). A Multi-Task CNN Learning Model for Taxonomic Assignment of Human Viruses. BMC Bioinform..

[69] Rice P., Longden I., Bleasby A. (2000). EMBOSS: The European Molecular Biology Open Software Suite. Trends Genet..

[70] Mistry J., Chuguransky S., Williams L., Qureshi M., Salazar G.A., Sonnhammer E.L.L., Tosatto S.C.E., Paladin L., Raj S., Richardson L.J. (2021). Pfam: The Protein Families Database in 2021. Nucleic Acids Res..

[71] Wheeler T.J., Eddy S.R. (2013). Nhmmer: DNA Homology Search with Profile HMMs. Bioinformatics.

