# Supplementary material for: Utilizing the VirIdAl Pipeline to Search for Viruses in the Metagenomic Data of Bat Samples
Source: Viruses. 2021 Oct 6;13(10):2006. doi: 10.3390/v13102006 (PMC8541124; doi:10.3390/v13102006)
Supplement: Supplementary file 1 [file viruses-13-02006-s001.zip › viruses-1319813-supplementary.pdf]

Supplementary materials

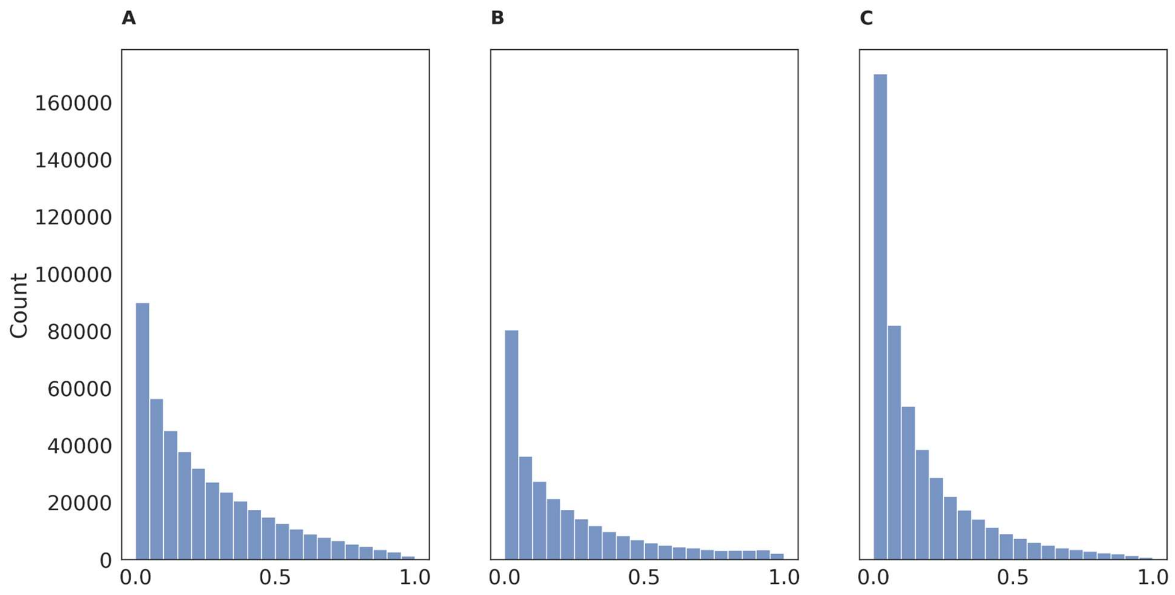

Supplementary figure S1. Distribution of the DeePaC-vir LSTM model scores assigned to unclassified sequences in samples 21 (A), 22 (B) and 33 (C).

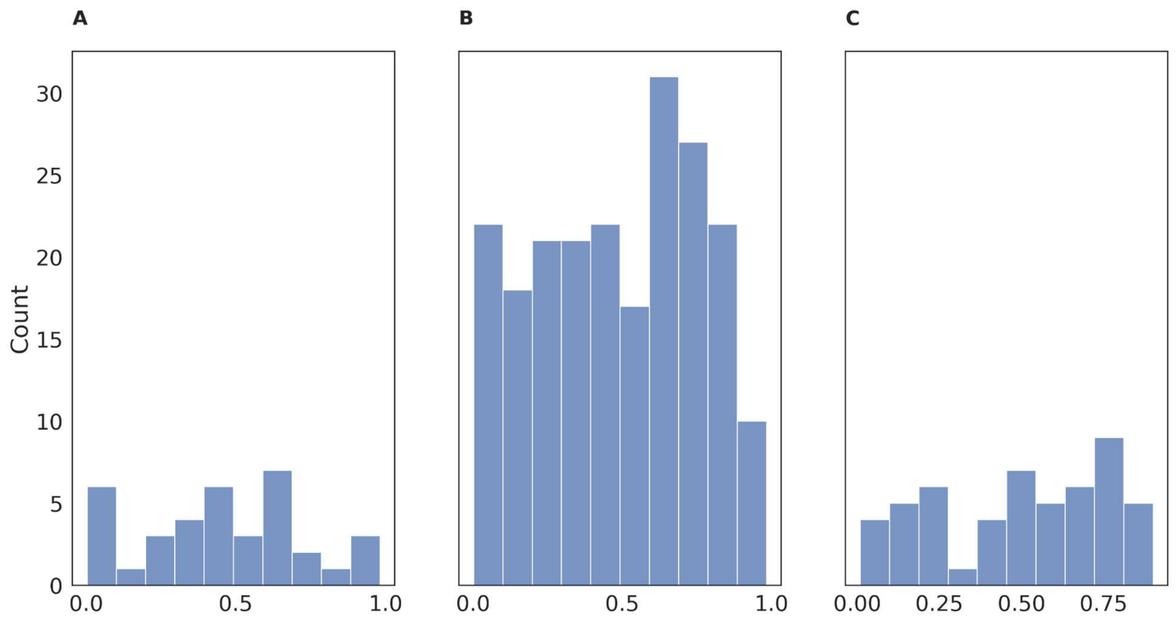

Supplementary figure S2. Distribution of the DeePaC-vir LSTM model scores assigned to *Betacoronavirus* sequences from samples 21 (A), 22 (B), and 33 (C).
